# The sequence kernel association test for the proportional odds model

**DOI:** 10.1093/bioinformatics/btaf304

**Published:** 2025-06-23

**Authors:** Jingxin Yan, Xiaoyu Zhang, Shuying Wang, Jinjuan Wang, Qizhai Li

**Affiliations:** State Key Laboratory of Mathematical Sciences, Academy of Mathematics and Systems Science, Chinese Academy of Sciences, Beijing 100190, China; School of Mathematical Sciences, University of Chinese Academy of Sciences, Beijing 100049, China; State Key Laboratory of Mathematical Sciences, Academy of Mathematics and Systems Science, Chinese Academy of Sciences, Beijing 100190, China; School of Mathematics and Statistics, Changchun University of Technology, Changchun 130012, China; School of Mathematics and Statistics, Beijing Institute of Technology, Beijing 100081, China; State Key Laboratory of Mathematical Sciences, Academy of Mathematics and Systems Science, Chinese Academy of Sciences, Beijing 100190, China; School of Mathematical Sciences, University of Chinese Academy of Sciences, Beijing 100049, China

## Abstract

**Motivation:**

The Sequence Kernel Association Test (SKAT) and its extensions are the most popular methods for studying the association between phenotypes and a set of single nucleotide polymorphisms. Their practical application is very wide, but most of these methods are designed for continuous and binary phenotypes. Ordered categorical phenotypes are also very common in practice, so there is an urgent need to develop SKAT-type tests for proportional odds model.

**Results:**

To accommodate ordered categorical phenotypes, we propose a test named the Sequence Kernel Association Test for the Proportional Odds Model (POM-SKAT). It constructs a score test for the variance of the coefficients of interest using a quasi-likelihood and the *P*-value is evaluated by approximating the asymptotic distribution of the test statistic with the Pearson Type III distribution. Simulation studies demonstrate that our method performs well and achieves high power in detecting gene-phenotype associations. We apply POM-SKAT to rheumatoid arthritis data provided by Genetic Analysis Workshop 16, identifying multiple relevant gene variants.

**Availability and implementation:**

Code is available at GitHub (https://github.com/amss-stat/POM-SKAT).

## 1 Introduction

Identifying the relationship between genes and diseases is an important topic in modern genetic studies, which has a profound significance for disease prevention and treatment. Many genetic associations with disease have been identified. For example, alleles in FGFR2 are associated with breast cancer (Hunter *et al.* 2007), Ahn *et al.* (2010) found single nucleotide polymorphism (SNP) rs2282679 associated with circulating vitamin D levels, and the genomic region 11p11.2 is associated with schizophrenia (Yue *et al.* 2011). The association between genes and diseases, combined with proteomics technology and information about the patient’s lifestyle, biomarkers and other factors, can provide patients with more effective and safer personalized treatment. For example, the widely used targeted therapies in oncology, and genetic screening in individuals with a family history of certain hereditary diseases, enable preventive measures to reduce disease risk.

In genome-wide association studies, the genetic association is typically examined by regressing the phenotype onto each individual SNP, and a parametric *P*-value is generated. However, these tests require strict thresholds, and polygenic and epistatic effects are often present. A number of methods, which can improve the effectiveness of the tests, have been proposed to test the association between gene sets and diseases. Commonly used testing methods include principal component analysis (PCA) (Gauderman *et al.* 2007), which projects SNPs onto a new coordinate system, maximizes variance through linear transformation; minimum *P*-value (MinP), which first tests individual SNP, then takes the smallest *P*-value and uses its empirical distribution to find the *P*-value for a multi-SNP test; The Sequence Kernel Association Test (SKAT) (Wu *et al.* 2010) and its variants, which construct test statistics by combining test statistics corresponding to individual gene variants. Variants of SKAT can handle various types of data. For example, Zhao *et al.* (2015) proposed extended SKAT for dealing with gene–environment interactions, and Zhang *et al.* (2020) proposed Composite Kernel Association Test (CKAT), which deals with gene and treatment interactions. Ionita-Laza et al. (2013), Chen *et al.* (2013), and Saad and Wijsman (2014) extended the SKAT approach by various methods to deal with data collected from families. Turkmen and Lin (2021) further extended SKAT to data related to the X chromosome. Wu and Pankow (2016) and Zhan *et al.* (2017) extended SKAT to analyze multiple phenotypes simultaneously.

Although SKAT and its variants have been well developed in the literature, they are specially designed for continuous and dichotomous phenotypes, while multi-category or ordered categorical phenotypes are often encountered in practice. For example, liver steatosis has three levels, consisting of normal liver, intermediate steatosis, and severe steatosis. The anti-cyclic citrulline peptide (anti-CCP) categorizes four ordered levels of risk for developing rheumatoid arthritis (RA) (Xue *et al.* 2019). Bocher *et al.* (2021) reformulated the SKAT test statistic as a weighted sum of the distance between the case center and the sample center, and then extend it to multi-category phenotypes without incorporating covariates. Beyond that we find no other extended SKAT for dealing with multi-category phenotypes within the limits of our search. To fill up this gap, we propose the sequence kernel association test for proportional odds model (POM-SKAT) by combining SKAT with the proportional odds model (POM).

Our POM-SKAT uses the POM to model the relationship between the ordered categorical variable and the explanatory variable and adopts the method in Lin (1997) to construct a score test statistic similar to SKAT. Our approach has three advantages. Firstly, we use the variance component test to convert multiple parameter tests into a single variance parameter test, which simplifies the testing problem. Moreover, we use quasi-likelihood to reduce the model complexity and computational burden for discrete variables, as directly conducting the score test involves different categories and individuals. Finally, we construct a SKAT-type test statistic that follows a mixed chi-square distribution, and its *P*-value can be found by re-sampling. Here we use the Pearson Type III distribution to approximate it, as its three parameters have explicit expressions, allowing for faster calculations.

We test our approach with data simulations and real data analysis. In the simulation, we construct 18 models under the null hypothesis by varying the number of phenotype categories, the number of considered SNPs, and the correlation among SNPs. We generate the Q–Q plots for all these models, and the results show that the distribution of our test statistic is well approximated by the Pearson Type III distribution. To evaluate the power of our method, we compare it with PCA, Bonferroni correction (BC) and Quadratic test statistic (QT) under the conditions that the number of associated SNPs ranges from 1 to 8. We further explore the scenario in which gene–gene interactions exist. The results show that our method has higher power. Our method together with MinP, BC and PCA are used to analyze some RA data provided by Genetic Analysis Workshop 16 (Amos *et al.* 2009), which verified the practicability of our method.

## 2 Materials and methods

Suppose that in a genetic association study, *n* independent individuals are randomly selected from a source population. For each i∈{1,2,…,n}, let $y_{i}\in \{0,\ldots ,J-1\}$ represent an ordered categorical phenotype with a total of *J* categories. $x_{i}={\left(x_{i1},\ldots ,x_{iK}\right)}^{⊤}$ is a vector of *K* covariates, and $g_{i}={\left(g_{i1},\ldots ,g_{iM}\right)}^{⊤}$ is a vector of *M* genotypes, where $g_{im}\in \{0,1,2\}$ denotes the number of *m*-th minor alleles of the genetic variant for the *i*-th individual. To model the relationship between $y_{i}$, $x_{i}$, and $g_{i}$, the POM is employed, which is expressed as

(1) $$\begin{array}{l} P(y_{i}=0)=\frac{1}{1+\text{exp}\;\{\alpha_{0}+g_{i}^{⊤}\beta +x_{i}^{⊤}\gamma \}},\\ P(y_{i}=j)=\frac{1}{1+\text{exp}\;\{\alpha_{j}+g_{i}^{⊤}\beta +x_{i}^{⊤}\gamma \}}\\ -\frac{1}{1+\text{exp}\;\{\alpha_{j-1}+g_{i}^{⊤}\beta +x_{i}^{⊤}\gamma \}},\\ j=1,\ldots ,J-2,\\ P(y_{i}=J-1)=1-\frac{1}{1+\text{exp}\;\{\alpha_{J-2}+g_{i}^{⊤}\beta +x_{i}^{⊤}\gamma \}}, \end{array}$$

where $\beta ={\left(\beta_{1},\ldots ,\beta_{M}\right)}^{⊤}$ and $\gamma ={\left(\gamma_{1},\ldots ,\gamma_{K}\right)}^{⊤}$ represent the coefficients for $g_{i}$ and $x_{i}$, respectively. To test the association between phenotype $y_{i}$ and the genotypes $g_{i}$, the following methods are proposed.

### 2.1 Principal component analysis

PCA is a commonly used dimensionality reduction method. Gauderman *et al.* (2007) applied it to examine the association between multiple SNPs and binary phenotype, but its availability of ordered categorical phenotypes has not been studied. To apply this method to ordered categorical phenotypes, we first perform a PCA for *M* SNPs. Assume that the first *L* principal components collectively capture 80% of the variability in the *M* genotypes. These components can be expressed as

$$\begin{array}{c} \psi_{i1}=e_{i1}^{⊤}g_{i}=e_{i,11}g_{i1}+e_{i,12}g_{i2}+\cdots +e_{i,1M}g_{iM},\\ \psi_{i2}=e_{i2}^{⊤}g_{i}=e_{i,21}g_{i1}+e_{i,22}g_{i2}+\cdots +e_{i,2M}g_{iM},\\ \vdots \\ \psi_{iL}=e_{iL}^{⊤}g_{i}=e_{i,L1}g_{i1}+e_{i,L2}g_{i2}+\cdots +e_{i,LM}g_{iM} \end{array}$$

where $e_{il}^{⊤}e_{il}=1,l=1,\ldots ,L$ are the eigenvectors of the correlation matrix of $g_{i}$, and the variances of $\psi_{il},l=1,\ldots ,L$, correspond to the eigenvalues in decreasing order. Then we construct POM using $\psi_{i}={\left(\psi_{i1},\ldots ,\psi_{iL}\right)}^{⊤}$, which can be expressed as

$$\begin{array}{c} P(y_{i}=0)=\frac{1}{1+\text{exp}\;\{\alpha_{0}+\psi_{i}^{⊤}b+x_{i}^{⊤}\gamma \}},\\ P(y_{i}=j)=\frac{1}{1+\text{exp}\;\{\alpha_{j}+\psi_{i}^{⊤}b+x_{i}^{⊤}\gamma \}}\\ -\frac{1}{1+\text{exp}\;\{\alpha_{j-1}+\psi_{i}^{⊤}b+x_{i}^{⊤}\gamma \}},\\ y=1,2,\ldots ,J-2,\\ P(y_{i}=J-1)=1-\frac{1}{1+\text{exp}\;\{\alpha_{J-2}+\psi_{i}^{⊤}b+x_{i}^{⊤}\gamma \}}. \end{array}$$

To test the null hypothesis $H_{0}:b_{1}=\cdots =b_{L}=0$ in this method, the likelihood ratio test (LRT) is applied. The resulting test statistic asymptotically follows a chi-square distribution with *L* degrees of freedom.

### 2.2 Minimum *P*-value

To obtain the minimum *P*-value, we first test each SNP individually. Specifically, for each genotype *m*, we construct the POM model as follows, independently,

$$\begin{array}{c} P(y_{i}=0)=\frac{1}{1+\text{exp}\;\{\alpha_{0}+g_{im}\beta_{m}+x_{i}^{⊤}\gamma \}},\\ P(y_{i}=j)=\frac{1}{1+\text{exp}\;\{\alpha_{j}+g_{im}\beta_{m}+x_{i}^{⊤}\gamma \}}\\ -\frac{1}{1+\text{exp}\;\{\alpha_{j-1}+g_{im}\beta_{m}+x_{i}^{⊤}\gamma \}},\\ j=1,\ldots ,J-2,\\ P(y_{i}=J-1)=1-\frac{1}{1+\text{exp}\;\{\alpha_{J-2}+g_{im}\beta_{m}+x_{i}^{⊤}\gamma \}}, \end{array}$$

and use the Student’s *t* test to get the *P*-value, $p_{m}$ for each SNP. The minimum *P*-value is then defined as $\text{minp}=\min\{p_{1},\ldots ,p_{M}\}$. To calculate the final *P*-value for the multi-SNP test, we employ a permutation approach. Specifically, within each permutation *r*, we randomly shuffle the phenotype data and calculate the corresponding minimum *P*-value ${\text{minp}}_{r}^{*}$. Using *R* random permutations to account for the null distribution, we obtain the empirical distribution of minimum *P*-values. The *P*-value of the multi-SNP test is then calculated as the proportion of permutation minimum *P*-values that are less than or equal to the observed minimum *P*-value,

$$\text{MinP}=\frac{1+\sum_{r=1}^{R}I({\text{minp}}_{r}^{*}\leq \text{minp})}{R+1}.$$

The BC is another commonly used method for multiple testing. It allocates a fraction of the overall significance level to each individual test, such that the significance level for each test becomes 1/M of the overall level. Consequently, the *P*-value for multiple testing is adjusted as M×minp.

### 2.3 POM-SKAT

We first use the variance component test to simplify the test problem, assuming that γ is constant and β follows a distribution with mean $0_{M}$ and variance D(θ) (Lin 1997). Here $0_{M}$ is a vector with all zeros and D(θ) is a linear function of θ, such that D(θ)=0 when θ=0, and that the third and higher-order moments of β are of order o(||θ||). Thus, determining whether there are correlations between the phenotype and the genotypes is equivalent to testing whether the variance of β is zero, that is, testing the null hypothesis $H_{0}:\beta_{1}=\cdots =\beta_{M}=0$ corresponds to testing $H_{0}:\theta =0$.

As derived in the Appendix (Derivation of POM-SKAT test statistics), the test statistic of our method is given by:

(2) $$T=\frac{1}{2}{\left(y-\overset{⁁}{\mu }\right)}^{⊤}\tilde{G}D^{\prime }(\theta ){\tilde{G}}^{⊤}(y-\overset{⁁}{\mu }),$$

where $y={\left(y_{1},\ldots ,y_{n}\right)}^{⊤}$ is the phenotype of the *n* individuals, $\overset{⁁}{\mu }={\left({\overset{⁁}{\mu }}_{1},\ldots ,{\overset{⁁}{\mu }}_{n}\right)}^{⊤}$ is the maximum likelihood estimator of expectation μ under $H_{0}$, defined as

$$\begin{array}{c} {\overset{⁁}{\mu }}_{i}=\sum_{j=0}^{J-1}\frac{\;\text{exp}\;\{{\overset{⁁}{\alpha }}_{j}+x_{i}^{⊤}\overset{⁁}{\gamma }\}}{1+\text{exp}\;\{{\overset{⁁}{\alpha }}_{j}+x_{i}^{⊤}\overset{⁁}{\gamma }\}}, \end{array}$$

and

(3) $$\begin{array}{c} {\left({\overset{⁁}{\alpha }}^{⊤},{\overset{⁁}{\gamma }}^{⊤}\right)}^{⊤}=\underset{\alpha ,\gamma }{\arg\min}\prod_{i=1}^{n}\prod_{j=0}^{j=J-1}{\left[P(y_{i}=j)\right]}^{I\{y_{i}=j\}}\\ s.t.\;{\overset{⁁}{\alpha }}_{0}<{\overset{⁁}{\alpha }}_{1}<\cdots <{\overset{⁁}{\alpha }}_{J-2} \end{array}$$

the specific solution methods for this optimization problem can be found in Agresti (2010) and McCullagh (1980). Here we directly use the polr() in the MASS package of R to get the solution. $\tilde{G}$ is the residual matrix from the regression of each genotype *m* onto x, which is used to account for the effects of gene-covariate associations. $D^{\prime }(\theta )$ is the derivative of D(θ), according to Wu *et al.* (2011), we take Beta(MAF, 1/2,1/2)^2^ as $D^{\prime }(\theta )$, where MAF is the minor allele frequency.

The statistic *T* follows a mixed chi-square distribution $\sum_{m=1}^{M}\lambda_{m}\chi_{m1}^{2}$, where $\chi_{m1}^{2},m=1,\ldots ,M$ are independent random variables following a chi-square distribution with one degree of freedom, and the mixed coefficient $\lambda ={\left(\lambda_{1},\ldots ,\lambda_{M}\right)}^{⊤}$ are the eigenvalues of $\frac{1}{2}V^{1/2}\tilde{G}D^{\prime }(\theta ){\tilde{G}}^{⊤}V^{1/2}$. Here V is a diagonal matrix with the variance of each $y_{i},i=1,\ldots ,n$ under $H_{0}$ on the diagonal. The *P*-value of $T^{*}$ can be approximated by re-sampling, but to increase the speed of calculation, we use Pearson Type III distribution P3(a,b,c) to approximate the distribution of $T^{*}$, where $T^{*}=\frac{T-E(T)}{\sqrt{\mathrm{Var}(T)}}$ is the standardized *T*.

Pearson Type III distribution (Pearson 1894) includes the normal distribution, the gamma distribution and other distributions, can be used to approximate various statistical distributions. According to Solomon and Stephens (1978), various methods for approximating the mixed chi-square distribution have been proposed, and many authors recommend using methods such as the Pearson III distribution to simplify calculations. Under the conditions of our article, the three parameters *a*, *b*, and *c* depend only on the skewness $\xi =\frac{E(T^{3})}{\mathrm{Var}{\left(T\right)}^{3/2}}$. Specifically, $a=\frac{\xi }{2},b={\left(\frac{2}{\xi }\right)}^{2}$, and $c=-\frac{2}{\xi }$. Moreover, the first three moments of the mixed chi-square distribution mentioned above can be easily calculated using the eigenvalues obtained from the eigen() function in R, where $\lambda =\text{eigen}(\frac{1}{2}V^{1/2}\tilde{G}D^{\prime }(\theta ){\tilde{G}}^{⊤}V^{1/2})\$\text{values}$ in R code. Thus $E(T)=\sum_{m=1}^{M}\lambda_{m},\mathrm{Var}(T)=2\sum_{m=1}^{M}\lambda_{m}^{2}$, and $E(T^{3})=8\sum_{m=1}^{M}\lambda_{m}^{3}$. Then, the *P*-value of our test statistic is calculated using the ppearsonIII() function from PearsonDS R package, that is, $p=1-\text{ppearsonIII}(T^{*},b,c,a)$.

## 3 Simulation studies

We conduct simulation studies to assess the accuracy of using the Pearson Type III distribution to approximate the asymptotic distribution of $T^{*}$ under the null hypothesis. Due to the computational intensity of MinP, we do not compare its power in the simulations. Its performance will be compared with other methods in the real data analysis.

### 3.1 Type I error

To evaluate the type I error of the proposed method, we generate genotypes under the null hypothesis, that is $\beta =0_{M}$. We use a two-step process to generate genotypes. First, we generate $z_{i}={\left(z_{i1},\ldots ,z_{iM}\right)}^{⊤}$ from a multivariate normal distribution with mean $0_{M}$ and covariance $\rho^{\left|i-j\right|}$, where *i* and *j* are the indices of the elements. Based on the MAF, *f*, we then calculate the two quantiles of the standard normal distribution corresponding to ${\left(1-f\right)}^{2}$ and $1-f^{2}$, dividing the sample space into three intervals. In the second step, the genotype count 0, 1, and 2 is then assigned based on the interval containing the sample. For example, if $f_{1}$ is the frequency of the first SNP, the two quantiles of the standard normal distribution corresponding to ${\left(1-f_{1}\right)}^{2}$ and $1-f_{1}^{2}$ are denoted as $Q_{1}$ and $Q_{2}$. If $z_{i1}\leq Q_{1}$, then $g_{i1}=0$; if $Q_{1}<z_{i1}\leq Q_{2}$, then $g_{i1}=1$; and if $z_{i1}>Q_{2}$, then $g_{i1}=2$. Here the MAF $f={\left(f_{1},\ldots ,f_{M}\right)}^{⊤}$ consists of *M* equally spaced values from the range [0.1, 0.45]. We set covariates $x_{i}={\left(x_{i1},x_{i2}\right)}^{⊤}∼N_{2}(0_{2},{\left(0.5^{\left|i-j\right|}\right)}_{2})$, and the error term $\epsilon_{i}∼\text{logistic}(0,1)$. The phenotype $y_{i}$ is generated using a process similar to genotype generation. Specifically, we first calculate the phenotype as $y_{i}=x_{i}^{⊤}\gamma +\epsilon_{i},i=1,\ldots ,n$, where $\gamma ={\left(0.5,0.8\right)}^{⊤}$. The ordered categorical phenotype is then obtained by truncating $y={\left(y_{1},\ldots ,y_{n}\right)}^{⊤}$ at the quantile threshold values $D={\left(d_{1},\ldots ,d_{J-1}\right)}^{⊤}$. Depending on *J*, *M* and ρ, we created 18 scenarios for our simulation. The different combinations are shown in Table 1, where J=3 or 5; M=50,100, or 200; and ρ=0.2,0.5 or 0.8. We set $D={\left(0.16,0.84\right)}^{⊤}$ and $D={\left(0.2,0.4,0.6,0.8\right)}^{⊤}$; n=2000 and n=3000, for J=3 and 5, respectively.

**Table 1.** Different models used under $H_{0}$.

	J=3, n=2000								
	J=5, n=3000								
*M*	50			50			50		
ρ	0.2	0.5	0.8	0.2	0.5	0.8	0.2	0.5	0.8

**Figure 1.**
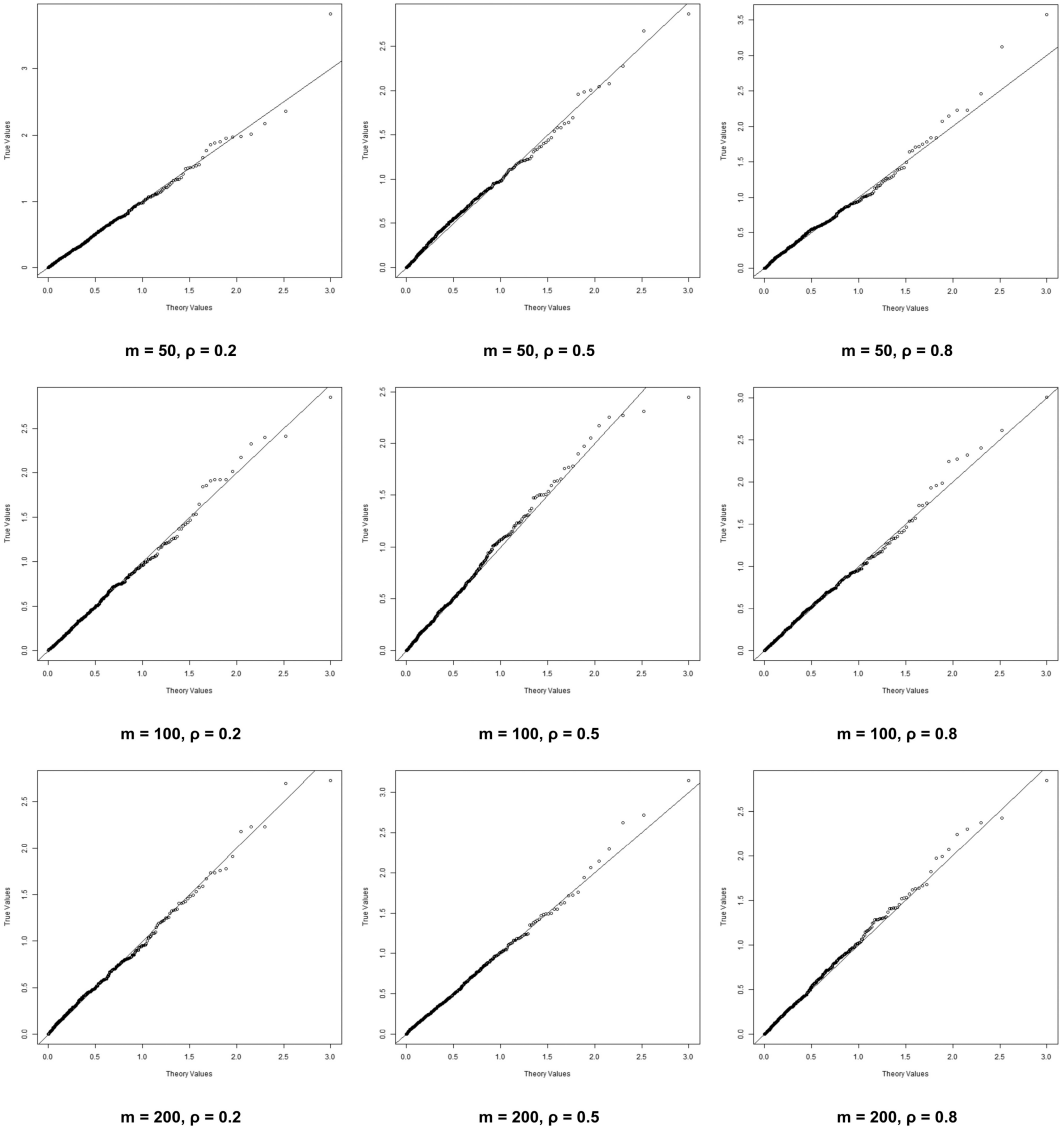
Q–Q plot of scenarios that J=3 and n=2000.

**Figure 2.**
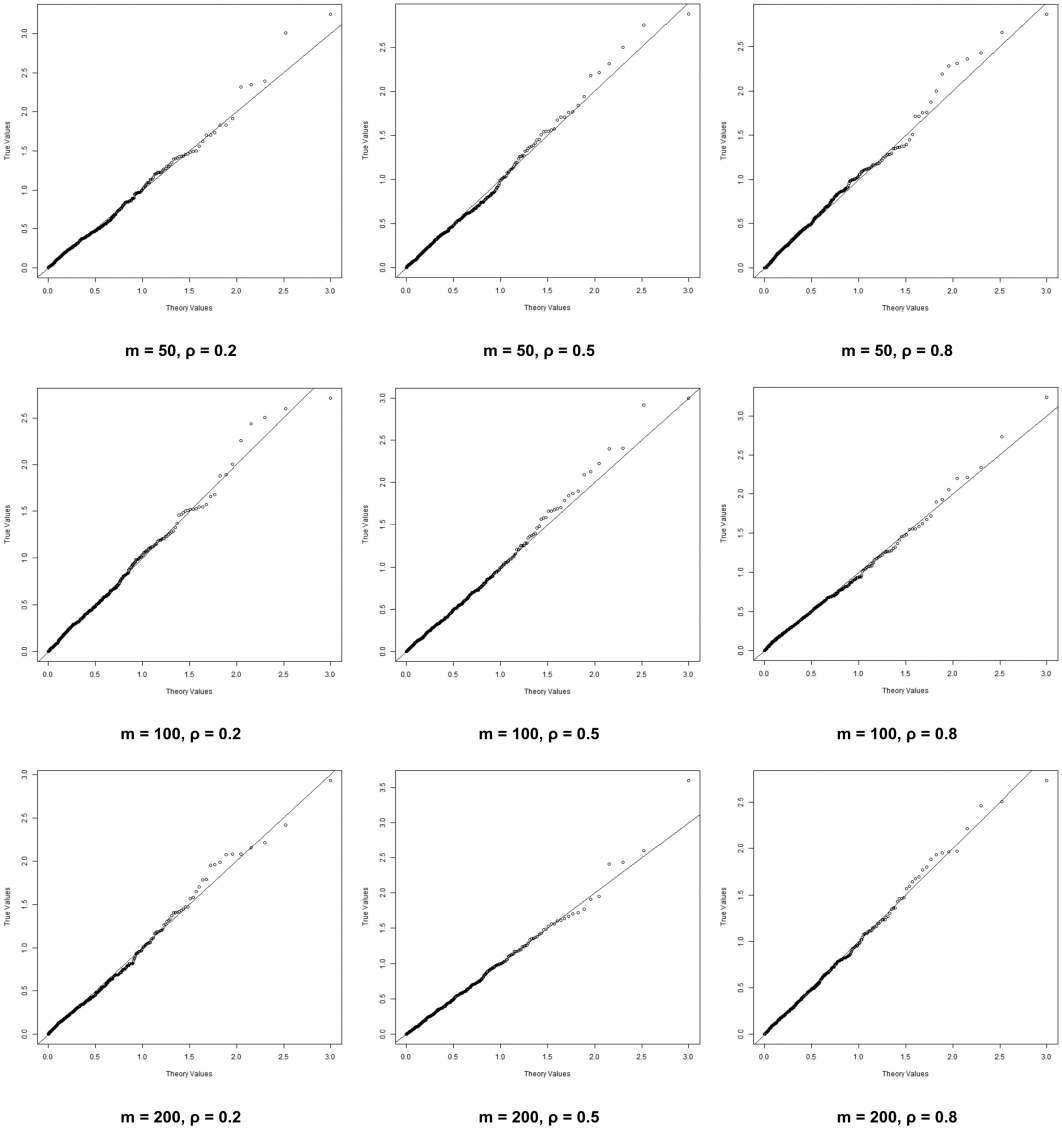
Q–Q plot of scenarios that J=5 and n=3000.

It is known that *P*-values follow the uniform distribution under the null hypothesis. To check the accuracy of our methods, we conduct 500 repeated experiments and plot the Q–Q plots comparing the theoretical and observed *P*-values. For a more detailed comparison, we apply a $-{\;\text{log}\;}_{10}$ transformation to the *P*-values and present the results in Figs 1 and 2. Figure 1 presents the results for J=3, while Fig. 2 corresponds to J=5. In both figures, the number of SNPs is fixed within each row, while the correlation between genotypes is fixed within each column. In each plot, the horizontal axis represents the theoretical *P*-values, while the vertical axis represents the observed *P*-values. In all Q–Q plots, the points are observed to almost fall on the diagonal, indicating that our simulation results are highly accurate. Table 7 in the appendices summarizes the Type I errors in the above scenarios, demonstrating desirable results. These results confirm the validity of our POM-SKAT and support the use of the Pearson Type III to approximate the null distribution.

### 3.2 Power

We generate data under the alternative hypothesis to compare the power of POM-SKAT with BC and PCA. To compare them with the classical method of hypothesis testing, we also construct a QT statistic for the results, which follows a chi-square distribution with degrees of freedom equal to the number of SNPs. The simulation settings are almost identical to those used under Type I Error analysis, except that we randomly select *M* SNPs from the CHODL genes, for which genotype data were provided by the Genetic Analysis Workshop 16, and we randomly select *B* SNPs and make them associated with phenotypes. Specifically, the phenotypes are first calculated as $y_{i}=x_{i}^{⊤}\gamma +g_{i}^{⊤}\beta +\epsilon_{i},i=1,\ldots ,n$, where $g_{i}={\left(g_{i1},\ldots ,g_{iB}\right)}^{⊤}$ and $\beta ={\left(\beta_{1},\ldots ,\beta_{B}\right)}^{⊤}$, and the ordered categorical phenotypes are then obtained by truncating $y_{i}$ using the same method as described earlier. We set M=30,60,90, choose *B* from 1 to 8, and fix $\beta_{b}=0.2,b=1,\ldots ,B$.

The power of the four methods is shown in Tables 2 and 3. The results show that the classical method fails when the number of SNPs is large, and its ability to control Type I error worsens as the number increases. For all other three methods, power increases as the number of SNPs associated with the phenotype (*B*) increases when the total number of SNPs (*M*) is fixed. Conversely, for a fixed *B*, power decreases as *M* increases. The power of our method is higher than the power of PCA and BC across nearly all scenarios. The advantage of POM-SKAT over PCA is more pronounced when the number of phenotypically associated SNPs is small. The advantage of POM-SKAT and PCA over BC is more pronounced when the number of phenotype-associated SNPs is large. For instance, for J=5,M=30,B=5, the powers of the POM-SKAT and PCA are 0.704 and 0.668, respectively, with a difference of 0.036. For J=5,M=90,B=7, the powers of the POM-SKAT and BC are 0.792 and 0.624, respectively, with a difference of 0.168. In addition, PCA has stronger requirements on sample size, and only when the sample size is large, PCA can better estimate the main component. Then, for fixed *n*, the advantage of POM-SKAT over PCA becomes more pronounced as *M* increases correspondingly.

**Table 2.** Power of POM-SKAT and PCA when J=3.

	M=30								
*B*	0	1	2	3	4	5	6	7	8
POM-SKAT	0.005	0.262	0.478	0.63	0.75	0.808	0.856	0.866	0.918
PCA	0.064	0.224	0.446	0.59	0.74	0.776	0.846	0.862	0.894
BC	0.05	0.306	0.478	0.57	0.658	0.662	0.742	0.786	0.81
QT	0.148	0.246	0.422	0.57	0.66	0.712	0.836	0.852	0.878

	M=60								
*B*	0	1	2	3	4	5	6	7	8
POM-SKAT	0.056	0.242	0.43	0.622	0.706	0.75	0.804	0.844	0.862
PCA	0.06	0.204	0.416	0.58	0.672	0.726	0.802	0.832	0.846
BC	0.042	0.238	0.4	0.558	0.586	0.62	0.664	0.736	0.718
QT	0.42	0.436	0.512	0.59	0.618	0.738	0.766	0.75	0.76

	M=90								
*B*	0	1	2	3	4	5	6	7	8
POM-SKAT	0.052	0.222	0.416	0.538	0.662	0.762	0.792	0.798	0.856
PCA	0.048	0.174	0.35	0.5	0.638	0.726	0.782	0.788	0.842
BC	0.018	0.246	0.358	0.456	0.548	0.63	0.684	0.654	0.704
QT	0.85	0.874	0.872	0.584	0.876	0.884	0.894	0.916	0.916

**Table 3.** Power of POM-SKAT and PCA when J=5.

	M=30								
*B*	0	1	2	3	4	5	6	7	8
POM-SKAT	0.048	0.186	0.328	0.48	0.586	0.704	0.788	0.826	0.88
PCA	0.05	0.164	0.286	0.446	0.58	0.668	0.774	0.816	0.866
BC	0.038	0.2	0.296	0.398	0.484	0.596	0.688	0.71	0.764
QT	0.144	0.192	0.296	0.43	0.554	0.638	0.754	0.85	0.876

	M=60								
*B*	0	1	2	3	4	5	6	7	8
POM-SKAT	0.042	0.164	0.306	0.476	0.518	0.656	0.71	0.814	0.86
PCA	0.05	0.134	0.288	0.416	0.506	0.636	0.698	0.81	0.844
BC	0.054	0.138	0.274	0.362	0.426	0.528	0.574	0.664	0.752
QT	0.402	0.442	0.474	0.558	0.620	0.652	0.708	0.794	0.848

	M=90								
*B*	0	1	2	3	4	5	6	7	8
POM-SKAT	0.042	0.15	0.292	0.388	0.518	0.636	0.704	0.792	0.848
PCA	0.06	0.124	0.272	0.36	0.486	0.604	0.682	0.768	0.822
BC	0.034	0.14	0.24	0.32	0.428	0.506	0.58	0.624	0.724
QT	0.88	0.86	0.872	0.874	0.9	0.894	0.906	0.922	0.952

To test whether our method is robust in the presence of gene–gene interactions, we perform additional simulations. The simulation setting is the same as in the above power simulations, except that we fix B=4 and add an interaction term between the first two SNPs. That is $y_{i}=x_{i}^{⊤}\gamma +g_{i}^{⊤}\beta +g_{i1}g_{i2}\phi +\epsilon_{i},i=1,\ldots ,n$, where $g_{i}={\left(g_{i1},\ldots ,g_{i4}\right)}^{⊤}$, $\beta ={\left(\beta_{1},\ldots ,\beta_{4}\right)}^{⊤}$ and ϕ=0.1. Then we truncate the values to obtain the ordered categorical phenotypes. Since QT does not perform well, we only simulate the other three methods. The results are shown in Table 4, which illustrates that POM-SKAT still outperforms PCA and BC.

**Table 4.** Power with gene interaction.

	J=3			J=5		
*M*	30	60	90	30	60	90
POM-SKAT	0.796	0.764	0.744	0.774	0.752	0.738
PCA	0.782	0.750	0.738	0.766	0.726	0.716
BC	0.72	0.706	0.678	0.724	0.702	0.654

### 3.3 Computation time

The Pearson Type III distribution is used to approximate the mixed chi-square distribution instead of re-sampling to save calculation time. To compare the calculation time of POM-SKAT and re-sampling, a simulation study was conducted. When simulate the computation time of POM-SKAT, the settings are similar to those used in the Type I error, except that the process is performed only once without repetition. For the re-sampling computation time, *H* permutations of Y is performed. In each permutation, the replaced Y and the original X and G are used to create a new sample and the value of *T* is re-evaluated using (2). Finally, the result from *H* permutations is used as the re-sampling p-value of the empirical distribution of *T*.

Simulations are performed with n=1000,2000,3000, and H=10000 under the condition J=3,ρ=0.5, and M=50 for re-sampling. The results, summarized in Table 5, demonstrate that the computation time required for POM-SKAT is significantly less than that for re-sampling, particularly for larger *n*. In these simulations, we conducted 10 000 permutations for each scenario. It is worth noting that there are theoretically n! possible phenotypic permutations. As *n* increases, the number of permutations required to achieve reliable results grows correspondingly, leading to a substantial increase in the computational burden for re-sampling methods.

**Table 5.** Computation time for POM-SKAT and re-sampling.

Method	POM-SKAT			Re-sampling		
*n*	1000	2000	3000	1000	2000	3000
Time	3 s	25 s	1 min 20 s	34 min 37 s	3 h 57 min 19 s	12 h 52 min 53 s

## 4 Real data analysis

RA has been shown to be a genetically linked disease. Anti-CCP is an effective predictor of RA. Higher anti-CCP levels are associated with an increased risk of developing RA. Specifically, anti-CCP level below 20, between 20 and 39, between 40 and 59, and over 60 corresponding to: without RA, low risk, moderate risk, and high risk. Studying the association between genetic variation and RA through its relationship with anti-CCP is a logical approach. Data form the Genetic Analysis Workshop 16 (Amos *et al.* 2009) is used for a genome-wide association analysis of RA, consisting of genotypes from 2062 individuals. Additionally, the dataset includes patients’ affection status with RA, sex, the anti-CCP titer and the genetic information. To identify genetic variants associated with RA using our method, we divide the sample into four categories based on the aforementioned anti-CCP criteria as response variables, each category contain 1195, 103, 66, and 698 samples, respectively. Since females are generally at higher risk of RA than males, sex is included as a covariate. We select six regions (Raychaudhuri *et al.* 2008, Lin *et al.* 2009) such as CD40, CHODL, and DGKB from the data as gene variation sets and test them separately with our method and three compared methods, the results are presented in Table 6. For MinP, 2000 permutations of anti-CCP are performed, while for PCA, the top 80% principal components are selected.

**Table 6.** Results of real data analysis.

Gene region name	*M*	POM	MinP	BC	PCA
CD40	31	0.0003	0.001	0.002	0.004
CHODL	98	0.195	0.27	0.503	0.072
DGKB	176	0.025	0.067	0.125	0.028
DNAH9	89	0.112	0.395	0.39	0.109
STAB2	89	0.0009	0.009	0.009	0.006
UST	126	0.111	0.27	0.5	0.073

**Table 7.** The empirical Type I error rates.

	m=50			m=100			m=200		
ρ	0.2	0.5	0.8	0.2	0.5	0.8	0.2	0.5	0.8
J=3,n=2000	0.058	0.046	0.058	0.046	0.052	0.044	0.052	0.056	0.048
J=5,n=3000	0.046	0.044	0.048	0.05	0.048	0.05	0.042	0.056	0.054

The results indicate that the conclusions are mostly consistent across methods, except for DGKB, where BC and MinP produce larger *P*-values, providing insufficient evidence to reject the null hypothesis under the significance level 0.05. However, the *P*-value of DGKB tested by POM-SKAT and PCA allow rejection of the null hypothesis at the same significance level. The *P*-values of CD40 and STAB2 are small, which means we can reject the null hypothesis, concluding that variations in these regions are associated with RA. And it can be seen that the *P*-value of our method is smaller when testing CD40 and STAB2, that is, 0.0003 and 0.0009 of our method, while MinP takes 0.001 and 0.009, BC takes 0.002 and 0.009, and PCA takes 0.004 and 0.006, meaning that our method is better at identifying relevant variants. The *P*-values of all four methods for CHODL, DNAH9, and UST are too big to reject the null hypothesis. Most of our results are consistent with the previous literature (Amos *et al.* 2009), which provides the data, indicates that CD40 has been implicated in Caucasian rheumatoid arthritis populations. Additionally, we identify DGKB and STAB2, which are recognized by Lin *et al.* (2009).

## 5 Discussion

We proposed the POM-SKAT to test the association between ordered categorical phenotypes and a set of SNPs, which is an extension of the widely used SKAT. To model the relationship between phenotypes and SNPs, we adopt the proportional odds model. As a multiple testing method, it involves testing all the coefficients of the SNPs in the set. To simplify this, we adopt the variance component test, which reduces the problem to testing only one parameter corresponding to their variance. To construct a score test statistic, for which only one parameter estimation is required under the null hypothesis, we use the quasi-likelihood. The Pearson Type III distribution, which can approximate any distribution by adjusting its three parameters, is employed to approximate the asymptotic distribution of the test statistic, saving computation time and simplifying calculations. Simulation results demonstrate that our POM-SKAT achieves good model fitting and high statistical power. The POM-SKAT shares a uniform form with SKAT and other extended SKAT methods. Similar to other SKAT test statistics, the central component (excluding the phenotypes and their expectations) can be seen as a kernel, which may be replaced by other kernels.

In our test statistic *T*, the $\left\{i,i^{\prime }\right\}$-th element of $\tilde{G}D^{\prime }(\theta ){\tilde{G}}^{⊤}$ is $\sum_{m=1}^{M}d_{m}^{\prime }g_{im}g_{i^{\prime }m}$, where $d_{m}^{\prime }$ is the {m,m}-th element of $D^{\prime }(\theta )$. This is equivalent to using a weighted linear kernel to process individual genetic data, which assumes that the effects of SNPs on phenotypes are independent of each other. We can handle SNP interactions by choosing other kernel functions. Many kernel functions have been proposed to handle SNP interactions, for example, the Gaussian kernel, identity by state (IBS) kernel and inner product-based kernel have been separately used by Wu *et al.* (2010) and Wang *et al.* (2023). However, it is worth noting that the performance and computational efficiency of different kernels vary under different SNP relationships. Under the current settings, the performance of other kernels is suboptimal.

Currently, the SNPs under study are common variants, but rare variants are also frequently studied, with extensive literature available on this topic. For example, Wu *et al.* (2011) extended the SKAT to test association between rare variants and binary phenotype. Lin *et al.* (2016) and Lim *et al.* (2020) increased consideration of gene–environment interaction. Oualkacha *et al.* (2013) took into account the data from families, Yan *et al.* (2015) even considered the longitudinal family data. Urrutia *et al.* (2015) and Posner *et al.* (2020) extended SKAT by handling multiple candidate kernels. Lee *et al.* (2017) considered cases with multiple phenotypes. However, methods to test the association between rare variants and ordered categorical phenotypes remind blank, presenting a potential area for future development. When rare variants are considered, the sample size increases significantly, leading to greater computational difficulty and longer computation time. SKAT and its extended methods often incorporate additional strategies when addressing rare variants, which presents another avenue for future research.

Pleiotropy is a common phenomenon in organisms, where a single gene usually affects multiple phenotypes. For example, from Ensembl (https://www.ensembl.org), we know that ASAH1 is associated with Farber disease and spinal muscular atrophy associated with progressive myoclonic epilepsy. O'Reilly *et al.* (2012) proposed MultiPhen, which constructed the likelihood ratio test using the proportional dominance model, to test the association between gene variants and continuous or binary phenotypes. But how to take ordered categorical phenotypes into consideration reminds unsolved. In this case, genotypes can be considered as ordered categorical variables, and the phenotypes are explanatory variables, which looks can be solved by our method. The difficulty is, in our POM-SKAT, genotypes only have three levels, but phenotypes can be continuous, binary or multi-level with or without order. The form and value of covariate matrix for phenotypes need to be studied and solved again.

## 6 Appendix

### 6.1 Derivation of POM-SKAT test statistic

Then the expectation and variance of $y_{i}$, i∈{1,2,…,n}, will be,

(4) $$E(y_{i})=\sum_{j=0}^{J-1}\frac{\;\text{exp}\;\{\alpha_{j}+g_{i}^{⊤}\beta +x_{i}^{⊤}\gamma \}}{1+\text{exp}\;\{\alpha_{j}+g_{i}^{⊤}\beta +x_{i}^{⊤}\gamma \}},$$

 

(5) $$\mathrm{Var}(y_{i})=E(y_{i})-{\left[E(y_{i})\right]}^{2}+\sum_{j=1}^{J-1}\frac{2j\;\text{exp}\;\{\alpha_{j}+g_{i}^{⊤}\beta +x_{i}^{⊤}\gamma \}}{1+\text{exp}\;\{\alpha_{j}+g_{i}^{⊤}\beta +x_{i}^{⊤}\gamma \}}.$$

We set $E(y_{i}|\beta )=\mu_{i}^{\beta },\mathrm{Var}(y_{i}|\beta )=V(\mu_{i}^{\beta })$, $\alpha ={\left(\alpha_{0},\alpha_{1},\ldots ,\alpha_{J-1}\right)}^{⊤}$, and $\eta_{i}=g_{i}^{⊤}\beta +x_{i}^{⊤}\gamma$ for simplicity. Thus, we have $E(y_{i})=h(\eta_{i})$, where *h* is a monotonically increasing function of $\eta_{i}$ and has the form

$$h(\eta_{i})=\sum_{j=0}^{J-1}\frac{\;\text{exp}\;\{\alpha_{j}+\eta_{i}\}}{1+\text{exp}\;\{\alpha_{j}+\eta_{i}\}},$$

which can be solved by the variance component score test (Lin 1997).

To test $H_{0}$, we first use the quasi-likelihood, and the integrated quasi-likelihood is

$$L(\theta ,\gamma ,\alpha )=\text{exp}\;l(\theta ,\gamma ,\alpha )=\int \;\text{exp}\;\{\sum_{i=1}^{n}l_{i}(\gamma ,\alpha ;\beta )\}dF(\beta ),$$

where

$$l_{i}(\gamma ,\alpha ;\beta )\propto \int_{y_{i}}^{\mu_{i}^{\beta }}\frac{\left(y_{i}-t\right)}{V(t)}dt.$$

Consider L(θ,γ,α) as the expectation of $\text{exp}\;\{\sum_{i=1}^{n}l_{i}(\gamma ,\alpha ;\beta )\}$, and take a quadratic expansion about $\sum_{i=1}^{n}l_{i}(\gamma ,\alpha ;\beta )$ at the truth β=0, we have

$$\begin{array}{c} L(\theta ,\gamma ,\alpha )=\text{exp}\;\{\sum_{i=1}^{n}l_{i}(\gamma ,\alpha ;0)\}(1\\ +\frac{1}{2}\text{tr}\{[(\sum_{i=1}^{n}\frac{\partial l_{i}(\gamma ,\alpha ;0)}{\partial \eta_{i}}g_{i})(\sum_{i=1}^{n}\frac{\partial l_{i}(\gamma ,\alpha ;0)}{\partial \eta_{i}}g_{i}^{⊤})\\ +\sum_{i=1}^{n}\frac{\partial^{2}l_{i}(\gamma ,\alpha ;0)}{\partial \eta_{i}^{2}}g_{i}g_{i}^{⊤}]D(\theta )\}+o(||\theta ||)), \end{array}$$

 

$$\begin{array}{c} l(\theta ,\gamma ,\alpha )=\sum_{i=1}^{n}l_{i}(\gamma ,\alpha ;0)\\ +\frac{1}{2}\text{tr}\{G^{⊤}[\frac{\partial l(\gamma ,\alpha ;0)}{\partial \eta }\frac{\partial l(\gamma ,\alpha ;0)}{\partial \eta^{⊤}}+\frac{\partial^{2}l(\gamma ,\alpha ;0)}{\partial \eta \partial \eta^{⊤}}]\mathrm{GD}(\theta )\}\\ +o(||\theta ||), \end{array}$$

where $\frac{\partial l(\gamma ,\alpha ;0)}{\partial \eta }$ is an n×1 vector with elements $\frac{\partial l_{i}(\gamma ,\alpha ;0)}{\partial \eta_{i}}$ and $\frac{\partial^{2}l(\gamma ,\alpha ;0)}{\partial \eta \partial \eta^{⊤}}$ is an n×n diagonal matrix with elements $\frac{\partial^{2}l_{i}(\gamma ,\alpha ;0)}{\partial \eta_{i}^{2}}$ on the diagonal. Let’s set Δ,W and $W_{0}$ be n×n diagonal matrices with elements

$$\begin{array}{c} \delta_{i}=h^{\prime }(\eta_{i}),\\ w_{i}={\left[V(\mu_{i})\right]}^{-1}\delta_{i}^{2},\\ w_{0i}=-w_{i}+e_{i}(G_{i}-\mu_{i}), \end{array}$$

where $\mu_{i}=E(Y_{i})$ and $e_{i}=V^{\prime }(\mu_{i}){\left(h^{\prime }(\eta_{i})\right)}^{2}+\frac{h^{\prime \prime }(\eta_{i})}{V(\mu_{i})}$.

Then, under $H_{0}$, the initial score test statistic will be

(6) $$\begin{array}{c} U=\frac{\partial l(\gamma ,\alpha_{0},\alpha_{1};0)}{\partial \theta }{|}_{\gamma =\overset{⁁}{\gamma },\alpha =\overset{⁁}{\alpha }}\\ =\frac{1}{2}\text{tr}\{[(\sum_{i=1}^{n}\frac{(y_{i}-{\overset{⁁}{\mu }}_{i}){\overset{⁁}{\delta }}_{i}g_{i}}{V({\overset{⁁}{\mu }}_{i})})(\sum_{i=1}^{n}\frac{(y_{i}-{\overset{⁁}{\mu }}_{i}){\overset{⁁}{\delta }}_{i}g_{i}^{⊤}}{V({\overset{⁁}{\mu }}_{i})})+\sum_{i=1}^{n}{\overset{⁁}{w}}_{0i}]D^{\prime }(\theta )\}\\ =\frac{1}{2}\{{\left(y-\overset{⁁}{\mu }\right)}^{⊤}{\overset{⁁}{\Delta }}^{-1}\overset{⁁}{W}GD^{\prime }(\theta )G^{⊤}\overset{⁁}{W}{\overset{⁁}{\Delta }}^{-1}(y-\overset{⁁}{\mu })\\ -\text{tr}({\overset{⁁}{W}}_{0})GD^{\prime }(\theta )G^{⊤}\}, \end{array}$$

According to Zhang and Lin (2003), the second part of (6) is unrelated to *Y*, and the first part of (6) follows a mixed chi-square distribution. Since the part related to Δ and W only affects the mixing coefficients, we can further simplify *U* to *T*.

Conflict of interest: The authors declare that they have no competing interests.

## Data Availability

The data were obtained through participation in Genetic Analysis Workshop 16. Due to privacy and policy considerations, the data cannot be made publicly available. The original publication related to these data is Amos *et al.* (2009), as cited in the references.

## References

[btaf304-B1] Agresti A. Analysis of Ordinal Categorical Data. Hoboken, NJ: John Wiley & Sons, 2010.

[btaf304-B2] Ahn J , Yu K, Stolzenberg-Solomon R et al Genome-wide association study of circulating vitamin D levels. Hum Mol Genet 2010;19:2739–45. 20418485 10.1093/hmg/ddq155 PMC2883344

[btaf304-B3] Amos CI , Chen WV, Seldin MF et al Data for Genetic Analysis Workshop 16 Problem 1, association analysis of rheumatoid arthritis data. BMC Proc 2009;3 Suppl 7:S2. 10.1186/1753-6561-3-s7-s2 PMC2795916 20018009

[btaf304-B4] Bocher O , Marenne G, Tournier-Lasserve E et al; FREX Consortium. Extension of SKAT to multi-category phenotypes through a geometrical interpretation. Eur J Hum Genet 2021;29:736–44. 33446828 10.1038/s41431-020-00792-8 PMC8110546

[btaf304-B5] Chen H , Meigs JB, Dupuis J. Sequence kernel association test for quantitative traits in family samples. Genet Epidemiol 2013;37:196–204. 23280576 10.1002/gepi.21703 PMC3642218

[btaf304-B6] Gauderman WJ , Murcray C, Gilliland F et al Testing association between disease and multiple SNPs in a candidate gene. Genet Epidemiol 2007;31:383–95. 17410554 10.1002/gepi.20219

[btaf304-B7] Hunter DJ , Kraft P, Jacobs KB et al A genome-wide association study identifies alleles in FGFR2 associated with risk of sporadic postmenopausal breast cancer. Nat Genet 2007;39:870–4. 17529973 10.1038/ng2075 PMC3493132

[btaf304-B8] Ionita-Laza I , Lee S, Makarov V et al Family-based association tests for sequence data, and comparisons with population-based association tests. Eur J Hum Genet 2013;21:1158–62. 23386037 10.1038/ejhg.2012.308 PMC3778346

[btaf304-B9] Lee S , Won S, Kim YJ et al; T2D-Genes Consortium. Rare variant association test with multiple phenotypes. Gen Epidemiol 2017;41:198–209. 10.1002/gepi.22021 PMC5340648 28039885

[btaf304-B10] Lim E , Chen H, Dupuis J et al A unified method for rare variant analysis of gene‐environment interactions. Stat Med 2020;39:801–13. 31799744 10.1002/sim.8446 PMC7261513

[btaf304-B11] Lin X. Variance component testing in generalised linear models with random effects. Biometrika 1997;84:309–26.

[btaf304-B12] Lin X , Lee S, Wu MC et al Test for rare variants by environment interactions in sequencing association studies. Biometrics 2016;72:156–64. 26229047 10.1111/biom.12368 PMC4733434

[btaf304-B13] Lin Y , Zhang M, Wang L et al Simultaneous genome-wide association studies of anti-cyclic citrullinated peptide in rheumatoid arthritis using penalized orthogonal-components regression. BMC Proc 2009;3 Suppl 7:S20. 20018010 10.1186/1753-6561-3-s7-s20 PMC2795917

[btaf304-B14] McCullagh P. Regression models for ordinal data. J R Stat Soc Series B Stat Methodol 1980;42:109–27.

[btaf304-B15] O'Reilly PF , Hoggart CJ, Pomyen Y et al MultiPhen: joint model of multiple phenotypes can increase discovery in GWAS. PLoS One 2012;7:e34861. 22567092 10.1371/journal.pone.0034861 PMC3342314

[btaf304-B16] Oualkacha K , Dastani Z, Li R et al Adjusted sequence kernel association test for rare variants controlling for cryptic and family relatedness. Genet Epidemiol 2013;37:366–76. 23529756 10.1002/gepi.21725

[btaf304-B17] Pearson K. Contributions to the mathematical theory of evolution. Philos Trans R Soc Lond 1894;185:71–110.

[btaf304-B18] Posner DC , Lin H, Meigs JB et al Convex combination sequence kernel association test for rare‐variant studies. Genet Epidemiol 2020;44:352–67. 32100372 10.1002/gepi.22287 PMC7205561

[btaf304-B19] Raychaudhuri S , Remmers EF, Lee AT et al Common variants at CD40 and other loci confer risk of rheumatoid arthritis. Nat Genet 2008;40:1216–23. 18794853 10.1038/ng.233 PMC2757650

[btaf304-B20] Saad M , Wijsman EM. Combining family‐and population‐based imputation data for association analysis of rare and common variants in large pedigrees. Genet Epidemiol 2014;38:579–90. 25132070 10.1002/gepi.21844 PMC4190076

[btaf304-B21] Solomon H , Stephens MA. Approximations to density functions using Pearson curves. J Am Stat Assoc 1978;73:153–60.

[btaf304-B22] Turkmen AS , Lin S. Detecting X‐linked common and rare variant effects in family‐based sequencing studies. Genet Epidemiol 2021;45:36–45. 32864779 10.1002/gepi.22352

[btaf304-B23] Urrutia E , Lee S, Maity A et al Rare variant testing across methods and thresholds using the multi-kernel sequence kernel association test (MK-SKAT). Stat Interface 2015;8:495–505. 26740853 10.4310/SII.2015.v8.n4.a8 PMC4698916

[btaf304-B24] Wang J , Long M, Li Q. A maximum kernel-based association test to detect the pleiotropic genetic effects on multiple phenotypes. Bioinformatics 2023;39;btad291. 37104737 10.1093/bioinformatics/btad291 PMC10174706

[btaf304-B25] Wu B , Pankow JS. Sequence kernel association test of multiple continuous phenotypes. Genet Epidemiol 2016;40:91–100. 26782911 10.1002/gepi.21945 PMC4724299

[btaf304-B26] Wu MC , Kraft P, Epstein MP et al Powerful SNP-set analysis for case-control genome-wide association studies. Am J Hum Genet 2010;86:929–42. 20560208 10.1016/j.ajhg.2010.05.002 PMC3032061

[btaf304-B27] Wu MC , Lee S, Cai T et al Rare-variant association testing for sequencing data with the sequence kernel association test. Am J Hum Genet 2011;89:82–93. 21737059 10.1016/j.ajhg.2011.05.029 PMC3135811

[btaf304-B28] Xue Y , Wang J, Ding J et al A powerful test for ordinal trait genetic association analysis. Stat Appl Genet Mol Biol 2019;18:20170066. 10.1515/sagmb-2017-0066 30685746

[btaf304-B29] Yan Q , Weeks DE, Tiwari HK et al Rare-variant kernel machine test for longitudinal data from population and family samples. Hum Hered 2015;80:126–38. 27161037 10.1159/000445057 PMC4940283

[btaf304-B30] Yue WH , Wang HF, Sun LD et al Genome-wide association study identifies a susceptibility locus for schizophrenia in Han Chinese at 11p11. 2. Nat Genet 2011;43:1228–31. 22037552 10.1038/ng.979

[btaf304-B31] Zhan X , Zhao N, Plantinga A et al Powerful genetic association analysis for common or rare variants with high-dimensional structured traits. Genetics 2017;206:1779–90. 28642271 10.1534/genetics.116.199646 PMC5560787

[btaf304-B32] Zhang D , Lin X. Hypothesis testing in semiparametric additive mixed models. Biostatistics 2003;4:57–74. 12925330 10.1093/biostatistics/4.1.57

[btaf304-B33] Zhang H , Zhao N, Mehrotra DV et al Composite kernel association test (CKAT) for SNP-set joint assessment of genotype and genotype-by-treatment interaction in pharmacogenetics studies. Bioinformatics 2020;36:3162–8. 32101275 10.1093/bioinformatics/btaa125

[btaf304-B35] Zhao G , Marceau R, Zhang D et al Assessing gene-environment interactions for common and rare variants with binary traits using gene-trait similarity regression. Genetics 2015;199:695–710. 25585620 10.1534/genetics.114.171686 PMC4349065

